# Does Self-Perceived Diet Quality Align with Nutrient Intake? A Cross-Sectional Study Using the Food Nutrient Index and Diet Quality Score

**DOI:** 10.3390/nu15122720

**Published:** 2023-06-12

**Authors:** Maximilian Andreas Storz

**Affiliations:** Center for Complementary Medicine, Department of Internal Medicine II, Freiburg University Hospital, Faculty of Medicine, University of Freiburg, 79106 Freiburg, Germany; maximilian.storz@uniklinik-freiburg.de

**Keywords:** diet quality, nutrient intake, total nutrient index, food nutrient index, diet quality score, National Health and Nutrition Examination Surveys

## Abstract

A reliable diet quality (DQ) assessment is critical to empower individuals to improve their dietary choices. Controversies persist as to whether self-perceived DQ is accurate and correlated with actual DQ as assessed by validated nutrient intake indexes. We used National Health and Nutrition Examination Surveys data to examine whether a higher self-perceived DQ was positively associated with a more optimal nutrient intake as reflected by the Food Nutrient Index (FNI) and Diet Quality Score (DQS). Comparative analyses were performed for three self-perceived DQ groups: (I) “excellent or very good” DQ, (II) “good or fair” DQ, and (III) “poor” DQ. The FNI and DQS differed substantially across groups and sexes. FNI scores ranged from 65 to 69 in participants with a self-reported excellent or very good DQ, whereas participants with a self-perceived poor DQ scored significantly lower (53–59). We also observed age- and sex-specific patterns, with the lowest overall FNI scores found in males aged 18–30 years and females aged 31–50 years. DQ intergroup differences were more pronounced in females than in males. Our findings suggest that higher self-perceived DQ is associated with a more optimal nutrient intake and indicate potential helpfulness of self-perceived DQ as a quick and still underexplored indicator with intrinsic limitations.

## 1. Introduction

A healthy lifestyle, including a diet centered around vegetables, whole grains, legumes, and fruits, is of paramount importance for optimal health [1,2], whereas a sedentary lifestyle and a diet high in saturated fats and processed sugars are major contributors to cardiovascular disease and many chronic disorders [3,4,5,6].

The prevalence of lifestyle-related chronic disease in the United States (U.S.) is growing and exerts substantial health and economic costs [7,8]. Health coaching as a lifestyle medicine process, and proactively inquiring about lifestyle behaviors and attitudes gains increasing importance for healthcare professionals [9,10,11]. Above all, a rigorous and effective diet quality (DQ) assessment is critical to empower individuals to improve their dietary choices [4,12,13].

Consumer’s DQ perceptions in the U.S. are potentially inaccurate, and previous research indicated that individuals tend to overrate their diet in relation to widely established dietary guidelines (such as the Dietary Guidelines for Americans) [13,14].

DQ perception is often related to nutritional knowledge, for which significant associations with DQ were observed [15,16]. Nevertheless, a self-perceived DQ is still an underexplored indicator [15], particularly in large and nationally-representative samples. Xue et al. reported a low agreement between the self-perceived DQ and actual (scoring metric assessed) DQ based on data from the US-based National Health and Nutrition Examination Survey (NHANES) [17]. However, their study was limited to a highly selective sample of cancer survivors and dates back to the years 2005–2014. A comparable study by Fanelli et al., which focused on individuals with diabetes, also dates back to the years 2007–2009 [18]. Newer studies in the general population are warranted as controversies persist as to whether a self-perceived DQ assessment is accurate and correlated with the actual DQ as assessed by validated DQ indexes [13].

Moreover, it remains virtually unknown whether a higher self-perceived DQ translates into a more optimal nutrient intake. Previous studies examining potential associations between the self-perceived and actual DQ used exclusively food-based indexes, such as the healthy eating index (HEI) [15,17,18]. To the best of our knowledge, nutrient-based indexes have not been examined in this context so far.

The assessment of nutrient exposures is critical for evaluating population-level adherence to dietary recommendations and to understand associations between diet and health [19]. Cowan et al. recently validated the Total Nutrient Index (TNI) as a useful tool to assess total micronutrient exposures of under-consumed micronutrients among US adults [19,20]. We used the supplement-free version of this newly developed index in conjunction with the Diet Quality Score (DQS) by Fitzgerald, Dewar, and Veugelers in order to examine whether a self-perceived DQ is associated with a micronutrient intake that is more closely aligned to current dietary guidelines [21].

In this study, we hypothesized that a higher self-perceived DQ is positively associated with a more optimal nutrient intake (as reflected by both aforementioned DQ scores) in the U.S. general population.

## 2. Materials and Methods

### 2.1. Study Population and Design

This cross-sectional analysis uses data from the NHANES—a program of studies designed to assess the health and nutritional status of adults and children in the U.S. [22,23]. The NHANES is a nationally representative survey and collects data from the non-institutionalized US population using a multistage stratified sampling technique to select participants [24]. NHANES data are released in 2 year cycles. Survey data is collected at in-home interviews and physical examinations performed in mobile examination centers [25].

A detailed description of the NHANES including its background, history, and methods may be obtained from the official NHNAES website [22], the survey content brochure [26], and NHANES-related publications in the field of nutrition [27,28,29,30,31].

### 2.2. Nutrient Intake Assessment

The NHANES dietary data included two 24-h dietary recalls collected using a computer-assisted dietary interview software program: the USDA’s automated multiple-pass method [31,32]. The first 24 h dietary recall was conducted as an in-person interview, and the second was administered by telephone 3–10 days later. Nutrient intake assessment and quantification has been described elsewhere in detail [32]. For this study, we used only data from participants with a reliable dietary recall status as assessed by the NHANES variable “DR1DRSTZ” (indicating the quality and completeness of a survey participant’s response to the dietary recall section). The present analysis was based on data from the first day of the dietary interview component. No modelling techniques were employed to estimate usual intakes over time.

As the study aimed to explore potential associations between self-perceived diet quality and validated diet quality scores, we only considered data from foods and beverages.

### 2.3. Self-Perceived Diet Quality

Self-perceived diet quality was assessed using data from the Diet Behavior and Nutrition section, which provides personal interview data on various nutrition related topics [33]. As part of this module, NHANES participants were asked the following question: “In general, how healthy is your overall diet?”. Potential answer options included “excellent”, “very good”, “good”, “fair”, and “poor”. For this analysis, we combined participants who replied with “excellent” or “very good”. Participants who had replied with “good” or “fair” were combined into a second group. Participants who refused to reply were excluded from the analysis.

### 2.4. The Diet Quality Score

The DQS was published in 2002 and aggregated 17 nutrients into an overall summary measure. The score has been described elsewhere in great detail [21]. In brief, the DQS includes the following nutrients: carbohydrate, fat, saturated fat, protein, thiamin, riboflavin, niacin, phosphorous, magnesium, iron, zinc, selenium, and vitamins A, B6, B12, C, and E. It was designed to assess the compliance with the Dietary References Intakes (DRIs) for the aforementioned nutrients. The authors assigned a value of 1 to each of the age- and gender-specific nutrient recommendations that were met. A value of 0 was assigned for nutrient intake recommendations that were not met. For carbohydrate intake, the authors used a range (50–60% of total energy intake) and assigned a value of 1 for intakes within the range and a value of 0 for intakes above or below this range. Fitzgerald, Dewar, and Veugelers then summed the values assigned to each nutrient, resulting in an overall score ranging from 0 to 17 points. The DQS did not include intakes from supplements. DRIs used in the DQS assessment may be found in Supplementary Table S1 [34]. To simplify this approach, we calculated the DQS in a sex-specific manner and neglected the very minor differences in DRI across age categories for a minority of its components (vitamin B6 and magnesium).

### 2.5. The Total Nutrient Index and Food Nutrient Index

The TNI was developed to assess total usual intakes of under-consumed micronutrients among the US population from foods, beverages, and dietary supplements relative to the recommended dietary intakes (RDA) and Adequate Intakes (AI) in the Dietary Guidelines for Americans [19,20]. The score and its scoring algorithm have been described elsewhere in great detail [20].

The TNI focuses on under-consumed micronutrients in the U.S. population and has been validated using NHANES data [19]. The TNI includes the following nutrients: calcium, magnesium, potassium, and choline as well as the vitamins A, C, D, and E. The score is scored from 0 to 100 and truncated at 100% of the respective standard [20].

Higher scores reflect intakes more closely aligned with the recommendations found in the Dietary Guidelines for Americans [14]. Each of the micronutrient components included in the score is weighted equally. Cowan et al. reported that the mean TNI score for U.S. adults (aged 19 years or older) in the 2011–2014 NHANES cycles was 75.4 points including supplements. Without supplements, it was substantially lower (69 points) [20]. For this study, we did not consider supplement usage as we focused exclusively on nutrient intakes from foods. This allowed for a better alignment with the question on self-perceived DQ. Since we considered intakes from food only, the term Food Nutrient Index (FNI), which was also coined by Cowan et al., appeared more appropriate [20]. The FNI was calculated identically to the TNI, yet without the nutrient contributions from supplements. The term FNI is used hereafter.

### 2.6. Inclusion and Exclusion Criteria

Only participants with a full dataset (including sociodemographic and nutrient intake data) were considered eligible. Participants that refused responses to any of the included questions were treated as missing cases. Individuals aged 19 years or younger were excluded from the present analysis.

### 2.7. Ethical Approval

NHANES protocols were approved by the NCHS Research Ethics Review Board [35,36]. Written informed consent was obtained from all participants.

### 2.8. Statistical Analysis

We performed the entire statistical analysis with Stata version 14 (StataCorp, College Stadion, TX, USA) and used appropriate NHANES sample weights to account for the complex, multistage, probability sampling design of the study program. We constructed a specific 6-year-weight for the employed dietary data [36] since we appended 3 consecutive NHANES cycles (2011–2012, 2013–2014, and 2015–2016) to increase the sample size for analyses stratified by population subgroups.

In a first instance, we performed descriptive statistics and examined sociodemographic sample characteristics by DQ category. We presented all data in accordance with the recommendations of West, Berglund, and Heeringa for applied survey data analysis [37] and took into account the most recent NCHS data presentation standards for NHANES data [38]. We described continuous variables with their mean and standard error in parentheses when data was normally distributed. Weighted subpopulation summary statistics as well as histograms and box plots were used to assess the data distribution. For categorical variables, we presented weighted proportions with their corresponding standard error. Hereby, we followed the recommendations of Parker et al. [38] and carefully assessed the reliability of each proportion using Korn–Graubard confidence intervals and associated statistics [39].

Multivariate linear regression analysis (followed by adjusted Wald tests) was used to test for potential differences in continuous variables. For categorical variables, we used Stata’s design-adjusted Rao–Scott test and explored potential associations between DQ category and sociodemographic variables. In addition, we assessed differences in the in the weighted proportions itself using Stata’s “lincom” command.

The FNI was computed in an age- and sex-specific manner [19], whereas the DQS was computed sex-specifically. We also used scatterplots and the Pearson product-moment correlation coefficient to examine potential associations between both diet quality indices. Afterwards, we ran multivariate linear regression models to predict the DQS from self-reported DQ category and age in both sexes (model 1). In an additional model (model 2), we also adjusted for race/ethnicity, marital status, educational level, and annual household income. Moreover, we ran multiple age- and sex-specific multivariate linear regression models to predict the FNI from self-reported DQ category and a series of covariates (race/ethnicity, marital status, educational level, and annual household income). Marginsplots were then used to graph statistics from fitted models for a better overview. We used a *p* value < 0.05 as a cutoff for statistical significance for all tests.

## 3. Results

**Figure 1:**
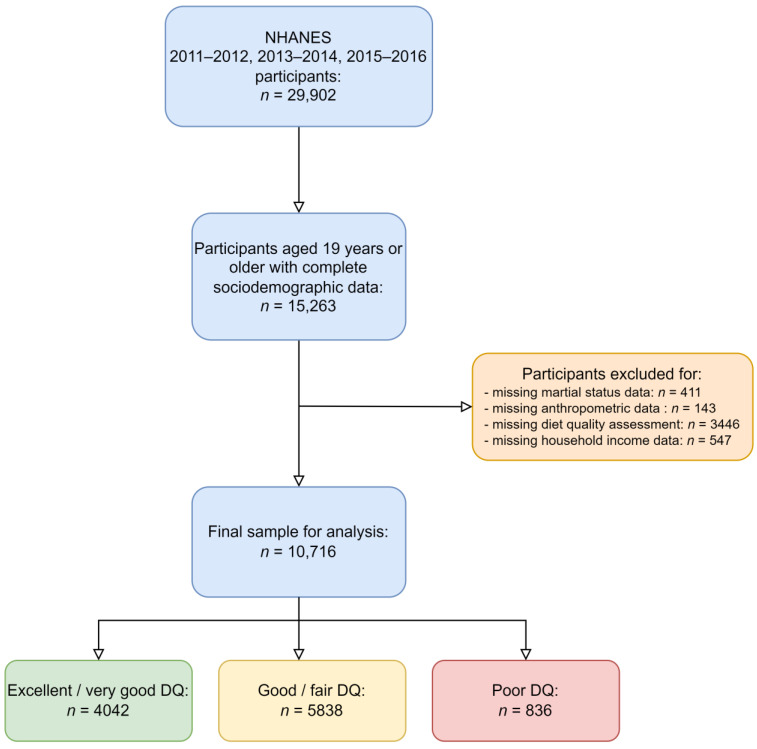
Participant inclusion flowchart.

The final sample for analysis included 10,716 participants after the exclusion of individuals with missing data and individuals not meeting the inclusion criteria (see Figure 1).

Table 1 shows sample characteristics by self-perceived DQ category. The three DQ groups did not differ significantly with regard to sex. Participants that indicated a poor DQ were significantly younger (mean: 43.14 years) than individuals with a good DQ (mean: 47.11 years) or very good/excellent DQ (mean: 51.49 years). Significant intergroup differences were also observed with regard to marital status, educational level, annual household income, and race/ethnicity. The weighted proportion of never married participants and widowed/divorced/separated participants in the poor DQ group was significantly higher compared to the other groups.

Self-perceived DQ was also associated with income. The proportion of individuals with an annual household income <$20,000 in the poor DQ group was almost twice as high as compared to the excellent DQ group. In addition to that, we observed a remarkably low proportion of individuals with a college degree or higher in the poor DQ group (11.64% vs. 30.34% and 44.10% in the other groups).

DQ was also associated with race/ethnicity. The weighted proportion of Mexican Americans and Non-Hispanic Blacks in the poor DQ group was approximately four times and two times higher, respectively, compared to the group with an excellent/very good DQ. Participants indicating an excellent DQ had a significantly lower BMI compared to the other groups. Notably, mean BMI in the first two DQ categories suggested overweight individuals, whereas individuals with a poor DQ were, on average, obese.

We found significant intergroup differences in total energy intakes across groups (Table 2). Participants indicating a poor DQ consumed, on average, 124 kcal more per day than individuals with an excellent or very good DQ. Despite these significant differences, we performed no energy-adjustments for nutrient intakes because we believe that energy intake differences across DQ groups are an intrinsic feature of this particular study sample. Significant intake differences were also found with regard to all three macronutrients. Fiber intake was highest in group 1 and lowest in participants with a poor DQ. The intakes across groups were statistically significant throughout for all examined nutrients except niacin, vitamin B12, calcium, iron, zinc, choline, and selenium.

**Figure 2:**
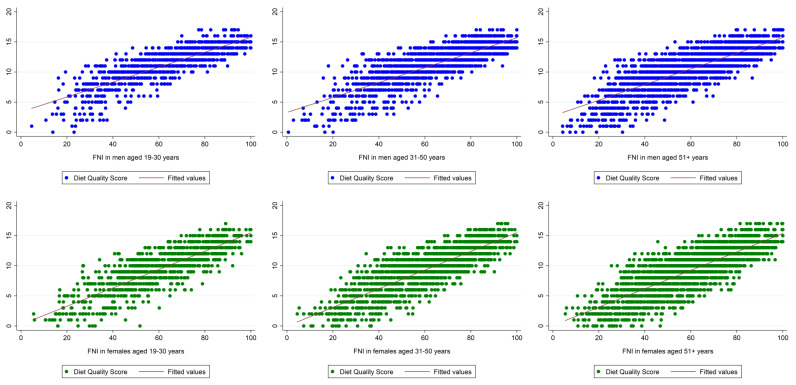
Weighted scatterplots of Diet Quality Scores (DQS) and Food Nutrient Index (FNI) scores in men and women by age group. Top: scatter plots (weighted) in men aged 19–30 years (**left**), 31–50 years (**middle**), and 51+ years (**right**). Bottom: scatter plots (weighted) in females aged 19–30 years (**left**), 31–50 years (**middle**), and 51+ years (**right**).

Table 3 shows crude DQS and FNI scores in males, whereas Table 4 shows both crude metrics in women. Both the DQS and FNI differed significantly across the three DQ groups. We found a significant linear trend for both (crude) metrics in both sexes, indicating higher scores in participants with an excellent/very good DQ and lower scores in participants with a poor DQ. Scatterplots (Figure 2) suggested a strong correlation between the DQS and the FNI across all age groups and in both sexes (Pearson’s product-moment correlation coefficients ranged from r = 0.81 to r = 0.84, *p* < 0.001 for all).

Table 5 and Table 6 show the final multivariate linear regression models examining the adjusted associations between DQ category and DQS in men and women. Covariates included age, race/ethnicity, marital status, educational level, and annual household income. Participants with a poor DQ and good/fair DQ yielded significantly lower DQS after adjustment for covariates. The differences appeared more pronounced in women (Table 6).

**Figure 3:**
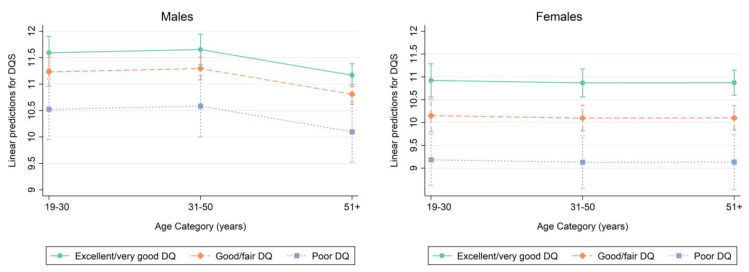
Marginsplot—Diet Quality Score (DQS) by self-perceived Diet Quality (DQ) category in men (**left**) and women (**right**). Plot of marginal predicted values for men based on the regression model 2 in Table 5, illustrating differences in the relationship of DQS and self-perceived DQ, depending on age category. Plot of marginal predicted values for women based on the regression model 2 in Table 6, illustrating differences in the relationship of DQS and self-perceived DQ, depending on age category.

Marginsplots were used to graph statistics from the fitted models. Marginal predicted values of DQS are shown in Figure 3 by DQ category for each age category in males on the left (based on model 2, Table 5) and females on the right (based on model 2, Table 6). Figure 3 suggests sex-specific differences in the relationship between self-perceived DQ and measured DQ as assessed by the DQS. The magnitude in age-specific DQS differences was more pronounced in women. 

**Figure 4:**
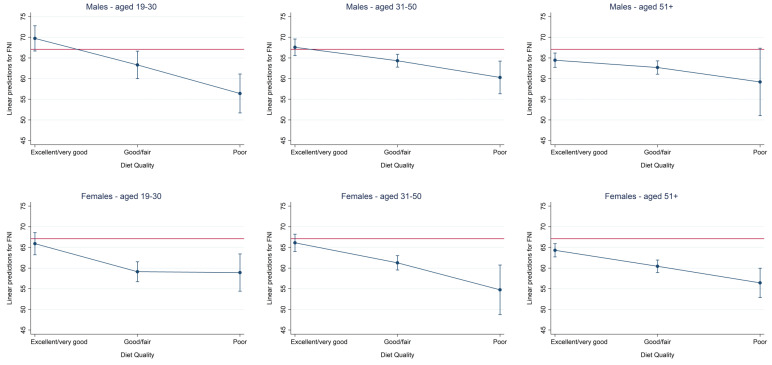
Marginsplot—Food Nutrient Index (FNI) by self-perceived Diet Quality (DQ) category in men (**top row**) and women (**bottom**). Top: Plot of marginal predicted values for men aged 19–30 years (**left**), 31–50 years (**middle**), and 51+ years (**right**) based on a multivariate linear regression model adjusting for race/ethnicity, educational level, marital status, and annual household income. Bottom: Plot of marginal predicted values for women aged 19–30 years (**left**), 31–50 years (**middle**), and 51+ years (**right**).

In a similar style, Figure 4 shows marginal predicted values for the FNI in an age- and sex specific manner after adjustment for race/ethnicity, educational level, marital status, and annual household income. For comparison, the red line indicates the mean TNI in 2011–2014 NHANES participants not taking any supplements as reported by Cowan et al. [19].

## 4. Discussion

The present cross-sectional study confirmed our hypothesis that a higher self-perceived DQ is positively associated with a more optimal nutrient intake as assessed by the FNI and DQS. A higher DQ was associated with older age and a lower BMI and lower total energy intake. The FNI and DQS differed substantially across self-perceived DQ groups. As for the DQS, differences across DQ categories were more pronounced in females. As for the FNI, age- and sex-specific patterns were observed, with lowest overall scores in males aged 19–30 years and females aged 31–50 years.

Diet is an important contributor to human health, and public health institutions are issuing guidelines aimed at improving healthy food choices [40,41]. Assessing DQ is essential for healthcare professionals when dealing with patients, yet due to lacking resources and insufficient time, many healthcare professionals neglect this important aspect of medicine [42,43,44].

Asking patients about their self-perceived DQ is a quick but also controversial way of gaining first information. Previous research indicated that individuals tend to overrate their diet in relation to widely established dietary guidelines [14]. For example, an older survey by Glanz, Bruck, and Assema conducted in the U.S. and the Netherlands revealed that participants in both countries lacked accurate awareness about how much fat they consumed [45].

Variyam, Shim, and Blaylock reported 20 years ago that approximately 40% of U.S. household meal planners judged their diet to be of a higher quality (in terms of healthfulness) than could be justified using a standard rubric (the HEI) [46]. As such, the authors cautioned that nutritionists and health professionals need to be aware of potential misperceptions and should alert dietary optimists about potentially false perceptions of their self-perceived diet quality.

Notably, both aforementioned studies date back to the late 1990s [45,46]. A more recent study by Rodrigues et al. revealed that a “good” self-perceived DQ was associated with the regular consumption of fruits and vegetables as well as high HEI scores in Brazilian adolescents [15]. Gregory, Smith, and Wendt reported that self-ratings of DQ were positively associated with household availability of dark green vegetables and low-fat milk and negatively associated with availability of sweetened soft drinks [13]. Based on these findings from the NHANES, it is not inconceivable that a higher self-perceived DQ translates into a more optimal nutrient intake profile.

Our study demonstrated exactly this and revealed positive associations between self-perceived DQ and two DQ metrics (the FNI and the DQS, which both are highly correlated as shown in Figure 2).

The differences in the DQ self-perception between males and females is noteworthy and warrants a further discussion. Previous studies suggested that men were more often unrealistic than women when it comes to a proper assessment of dietary intake [47]. Our results support these findings, and we thus caution healthcare professionals to be aware of potential misperceptions in men. Self-reported health has been associated with a healthier food consumption, improved nutrient intake, and more regular considerations of eating behaviors in various contexts [48,49,50], yet sex-specific aspects must always be taken into account.

Although our findings are based on nationally-representative data from the US, it remains questionable as to whether our results are generalizable to other populations outside the country. Previous studies revealed country- and culture-specific associations between a self-perceived DQ and objective DQ [51,52]. The association between a higher self-perceived DQ and increased vegetable consumption seems reproducible across studies [13,53], albeit observed in different populations. Nevertheless, it is difficult to classify and categorize our results in a comparative way as we are the first group to present associations with nutrient-based indexes.

Whether the DQS and FNI differences across DQ categories are clinically relevant also remains subject to debate. One could argue that differences of 1–2 points in DQS scores are potentially a minor finding. Then again, FNI differences of 5 points or more as shown in our study appear to be relevant when glancing at the recent study by Cowan et al. [19,20] and likely indicate substantial differences in nutrient intakes. Substantial differences in that range were found between individuals taking dietary supplements (DS) and individuals without DS intake. As DS consumption contributes substantially to nutrient intake in certain sociodemographic groups in the US [54,55], these differences are potentially of high significance. 

In light of the existing literature, our findings thus suggest that a self-perceived DQ is significantly associated with differences in nutrient intake in the general US population. Further studies in other populations that also include clinical endpoints for the FNI are warranted.

The present study has strengths and limitations that warrant further discussion. As for the strengths, we investigated a rarely discussed topic and made used of a recently developed nutrient-based DQ metric (the FNI), which has been shown to adequately assess micronutrient status in US adults. Our findings are based on a large sample size from the NHANES—a well-known nationally representative data source of high quality. The usage of an additional objective DQ metric (the DQS) is an additional asset. Although both indexes share a similar construction concept, they differ with regard to the amount of included nutrients. Some nutrients that are included in the FNI are not included in the DQS (e.g., choline) and vice versa. Moreover, the scoring system of the DQS (0 points for a recommendation that is not met, 1 point for a recommendation that is met) is somewhat restrictive as compared to the FNI, which is truncated at 100% of the respective standard and thus allows for a more nuanced nutrient intake assessment.

The age- and gender-specific analysis allows for new insights, and all analyses were performed in accordance with the most recent recommendations of West, Berglund, and Heeringa for applied survey data analysis [37].

As for the weaknesses, we deal with the intrinsic limitations of a cross-sectional study, which does not allow for causal interferences. Self-reported data (both questionnaire and dietary data) are subject to bias and prone to measurement errors. Validation studies for the TNI and the FNI (e.g., for construct validity) have not yet been published. The sample applies for studies using clinical endpoints. As specified earlier, we calculated the DQS in a sex-specific manner and intentionally neglected the very minor differences in DRI across age categories for a few of its components (namely vitamin B6 and magnesium). This could have introduced a minor loss of precision but made the study more feasibly in a way of reducing the total number of sub-analyses. Whether the observed intergroup differences in DQ metrics matter clinically was not ascertainable at the current point and will be subject to future research. Finally, it must be noted that the present analysis is based solely on data from the first day of the dietary interview component. No modelling techniques were employed to estimate usual intakes over time. This common approach has strengths and weaknesses itself that warrant a transparent discussion [56].

No matter which approach is employed, there is always some error associated with self-reported dietary intakes [32,57]. Traditionally, a single 24 h recall has been considered sufficient to describe mean dietary intakes and is useful for analytical and descriptive epidemiologic purposes [32,58]. On the other hand, Cowan et al. used modeling techniques to estimate usual nutrient intake distributions from foods and beverages in their original publication [19,20]. In this regard, we deviated from their approach and may have lost some precision in this process. On the one hand, several authors demonstrated that a single 24 h recall is sufficient to adequately estimate population means [19,59]. Based on these findings, and based on the fact that adding data from the second 24 h dietary recall would result in a substantial sample size reduction, we refrained from this step. Although Steinfeldt et al. recently demonstrated that mean energy intakes of NHANES adults were not statistically different between the two days of recall by sex, race/ethnicity, or income within selected age groups [60], we transparently acknowledge the limitations of our approach.

## 5. Conclusions

For the very first time, we reported associations between self-perceived DQ and objective DQ scores using a nutrient-based scoring system. Our findings reveal that a higher self-perceived DQ is associated with a more optimal nutrient intake (as reflected by significantly higher FNI scores). Higher self-perceived DQ was also associated with lower BMI and total energy intake. Our findings imply sex-specific associations, with more pronounced differences in women. The present results suggest potential helpfulness of self-perceived DQ as a quick and still underexplored indicator but also have some intrinsic limitations. Future studies that also include clinical endpoints are warranted.

## Figures and Tables

**Table 1:** Sample characteristics by diet quality category.

	Excellent/Very Good DQ *n =* 4042	Good/Fair DQ *n =* 5838	Poor DQ *n =* 836	*p* -Value
**Sex**				0.824 ^b^
Male	46.95% (0.84)	47.76% (0.89)	46.73% (3.01)	
Female	53.05% (0.84)	52.24% (0.89)	53.27% (3.01)	
**Age (years)**	51.49 (0.53)	47.11 (0.44)	43.14 (0.96)	<0.001 ^c^
**Marital status**				<0.001 ^b^
Married/Living with Partner	67.37 (1.47)	62.74% (1.27)	45.86% (2.64) ^e^	
Widowed/Divorced/Separated	17.34% (0.98)	18.03% (0.88)	26.45% (2.21) ^e^	
Never married	15.29% (1.12)	19.22% (1.33)	27.69% (2.33) ^e^	
**Annual household income**				<0.001 ^b^
<$20,000	11.42% (0.96)	14.73% (0.91)	22.28% (2.19) ^e^	
>$20,000	88.58% (0.96)	85.27% (0.91)	77.72% (2.19) ^e^	
**Education Level**				<0.001 ^b^
Less than 9th grade	2.86% (0.42)	4.39% (0.42)	6.09% (1.14) ^e^	
9–11th grade	6.80% (0.68)	9.17% (0.70)	17.91% (1.50) ^e^	
High school graduate/GED ^d^	16.47% (1.02)	21.97% (1.11)	27.30% (2.64) ^e^	
Some college or AA degree	29.77% (1.28)	34.13% (1.12)	37.08% (2.42) ^e^	
College graduate or above	44.10% (1.89)	30.34% (1.69)	11.64% (1.75) ^e^	
**Race/ethnicity**				<0.001 ^b^
Mexican American	3.63% (0.54)	7.86% (1.05)	13.56% (2.26) ^e^	
Other Hispanic	3.95% (0.49)	6.03% (0.78)	6.81% (1.28) ^e^	
Non-Hispanic White	73.85% (1.76)	67.46% (2.16)	57.02% (3.20) ^e^	
Non-Hispanic Black	8.65% (0.93)	10.47% (1.15)	16.33% (1.99) ^e^	
Other Race ^a^	9.92% (0.84)	8.18% (0.75)	6.27% (1.06) ^e^	
**BMI (kg/m^2^)**	27.24 (0.14)	28.97 (0.15)	32.98 (0.47)	<0.001 ^c^

Weighted proportions. Total number of unweighted observations: 10,716. Continuous variables shown as mean (standard error). Categorical variables shown as weighted proportion (standard error). All weighted proportions can be considered reliable, as peer recent NCHS Guidelines. ^a^ = includes Multi-Racial; ^b^ = based on Stata’s design-adjusted Rao–Scott test, ^c^ = based on regression analyses followed by adjusted Wald tests, ^d^ = or equivalent, ^e^ = indicates significant differences in the proportions.

**Table 2:** Nutrient and total energy intake by self-perceived diet quality category.

	Excellent/Very Good DQ *n =* 4042	Good/Fair DQ *n =* 5838	Poor DQ *n =* 836	*p*-Value
Total energy intake (kcal/d)	2085.88 (20.46)	2148.98 (16.65)	2210.01 (57.80)	0.038
Carbohydrate intake (g/d)	244.57 (2.50)	254.51 (2.31)	267.53 (8.59)	0.008
Carbohydrate intake (%tE)	47.31 (0.26)	48.00 (0.25)	48.85 (0.52)	0.008
Protein intake (g/d)	84.40 (1.09)	82.37 (0.61)	78.06 (2.13)	0.026
Protein intake (%tE)	16.60 (0.17)	15.75 (0.12)	14.51 (0.29)	0.026
Fat intake (g/d)	80.43 (1.04)	83.57 (0.83)	85.80 (2.40)	0.036
Fat intake (%tE)	34.13 (0.23)	34.43 (0.21)	34.34 (0.39)	0.036
Saturated fat intake (g/d)	25.31 (0.35)	27.19 (0.33)	28.48 (0.91)	<0.001
Saturated fat intake (%tE)	10.68 (0.09)	11.16 (0.0844)	11.31 (0.20)	<0.001
Fiber intake (g/d)	19.60 (0.32)	17.03 (0.20)	13.97 (0.49)	<0.001
Vitamin A intake (mcg RAE/d)	731.38 (16.52)	628.68 (22.74)	539.34 (34.09)	<0.001
Vitamin C intake (mg/d)	96.87 (2.64)	77.75 (2.12)	63.01 (3.66)	<0.001
Vitamin D intake (IE/d)	212.82 (7.06)	181.56 (3.07)	163.24 (12.83)	<0.001
Vitamin E intake (mg/d)	10.21 (0.21)	9.03 (0.12)	7.75 (0.27)	<0.001
Vitamin B1 intake (mg/d)	1.63 (0.02)	1.62 (0.01)	1.50 (0.05)	0.034
Vitamin B2 intake (mg/d)	2.26 (0.02)	2.15 (0.02)	2.094 (0.09)	0.003
Vitamin B3 intake (mg/d)	25.84 (0.27)	26.01 (0.25)	26.46 (1.01)	0.812
Vitamin B6 intake (mg/d)	2.25 (0.03)	2.11 (0.02)	2.10 (0.09)	0.002
Vitamin B12 intake (mcg/d)	5.19 (0.11)	5.07 (0.19)	5.33 (0.33)	0.783
Phosphorus intake (mg/d)	1425.97 (16.45)	1389.29 (8.91)	1340.44 (37.49)	0.047
Magnesium intake (mg/d)	338.49 (4.23)	300.62 (2.91)	267.35 (7.76)	<0.001
Potassium intake (mg/d)	2913.41 (33.74)	2639.44 (20.34)	2369.10 (65.74)	<0.001
Calcium intake (mg/d)	989.81 (15.07)	965.25 (10.17)	930.66 (32.99)	0.118
Iron intake (mg/d)	14.99 (0.17)	14.72 (0.15)	13.99 (0.60)	0.189
Zinc intake (mg/d)	11.41 (0.13)	11.27 (0.10)	10.52 (0.39)	0.075
Choline intake (mg/d)	291.58 (5.79)	290.23 (3.54)	287.99 (9.94)	0.952
Selenium intake (mcg/d)	118.31 (1.91)	114.73 (0.92)	108.51 (2.60)	0.111

Total number of unweighted observations: 10,716. Continuous variables shown as mean (standard error). The *p*-value is based on regression analyses followed by adjusted Wald tests.

**Table 3:** DQS and FNI in males by self-perceived diet quality category.

**Participants aged 19–30 years**	**Excellent/very good DQ** ***n = *** **319**	**Good/fair DQ** ***n = *** **539**	**Poor DQ** ***n = *** **99**	***p*** **-value**
DQS	11.77 (0.21)	11.13 (0.16)	10.30 (0.40)	0.004
FNI	68.93 (1.04)	62.21 (1.23)	54.63 (1.94)	<0.001
**Participants aged 31–50 years**	**Excellent/very good DQ** ***n = *** **566**	**Good/fair DQ** ***n = *** **934**	**Poor DQ** ***n = *** **160**	***p*** **-value**
DQS	11.86 (0.20)	11.29 (0.10)	10.47 (0.35)	<0.001
FNI	68.35 (1.15)	64.76 (0.65)	59.11 (2.04)	<0.001
**Participants aged 51+ years**	**Excellent/very good DQ** ***n = *** **1143**	**Good/fair DQ** ***n = *** **1300**	**Poor DQ** ***n = *** **110**	***p*** **-value**
DQS	11.19 (0.14)	10.86 (0.12)	9.66 (0.57)	0.025
FNI	65.13 (0.91)	62.47 (0.78)	56.30 (3.93)	0.029

**Table 4:** DQS and FNI in female by self-perceived diet quality category.

**Participants aged 19–30 years**	**Excellent/very good DQ** ***n = *** **300**	**Good/fair DQ** ***n = *** **639**	**Poor DQ** ***n = *** **103**	***p*** **-value**
DQS	11.33 (0.22)	9.90 (0.19)	9.42 (0.42)	<0.001
FNI	67.06 (1.34)	59.32 (1.08)	57.45 (2.09)	<0.001
**Participants aged 31–50 years**	**Excellent/very good DQ** ***n = *** **660**	**Good/fair DQ** ***n = *** **1033**	**Poor DQ** ***n = *** **177**	***p*** **-value**
DQS	11.13 (0.22)	10.24 (0.17)	8.79 (0.53)	<0.001
FNI	67.43 (1.02)	61.77 (0.88)	53.61 (2.78)	<0.001
**Participants aged 51+ years**	**Excellent/very good DQ** ***n = *** **1054**	**Good/fair DQ** ***n = *** **1393**	**Poor DQ** ***n = *** **187**	***p*** **-value**
DQS	10.84 (0.16)	9.98 (0.15)	8.46 (0.30)	<0.001
FNI	64.98 (0.84)	59.39 (0.76)	53.43 (1.75)	<0.001

**Table 5:** Multivariate linear regression models examining potential associations between DQ category and DQS in *n* = 5170 men.

Independent Variables	β	Linearized SE	*p*	β	Linearized SE	*p*
	Model 1			Model 2		
DQ						
Excellent/very good	-	-	-	-	-	-
Good/Fair	−0.46	0.12	<0.001	−0.36	0.12	0.005
Poor	−1.41	0.29	<0.001	−1.07	0.29	0.001

Significant regression equations were found for both models: F(4,44) = 9.22 (model 1) and F(15,33) = 10.82 (model 2), respectively, with a *p*-value < 0.001 for both and with R2 values of 0.018 and 0.050, respectively. The symbol “-” denotes the reference category. Model 1 adjusts for age, while Model 2 adjusts for age, race/ethnicity, marital status, educational level and annual household income.

**Table 6:** Multivariate linear regression models examining potential associations between DQ category and DQS in *n* = 5546 women.

Independent Variables	β	Linearized SE	*p*	β	Linearized SE	*p*
	Model 1			Model 2		
DQ						
Excellent/very good	-	-	-	-	-	-
Good/Fair	−0.97	0.13	<0.001	−0.77	0.12	<0.001
Poor	−2.24	0.31	<0.001	−1.74	0.29	<0.001

Significant regression equations were found for both models: F(4,44) = 20.62 (model 1) and F(15,33) = 19.25 (model 2), respectively, with a *p*-value < 0.001 for both and with R2 values of 0.030 and 0.063, respectively. The symbol “-” denotes the reference category. Model 1 adjusts for age, while Model 2 adjusts for age, race/ethnicity, marital status, educational level and annual household income.

## Data Availability

Data are publicly available online (https://wwwn.cdc.gov/nchs/nhanes/Default.aspx; accessed on 2 July 2022). The datasets used and analyzed during the current study are available from the corresponding author upon reasonable request.

## Supplementary Materials

The following supporting information can be downloaded at: https://www.mdpi.com/article/10.3390/nu15122720/s1, Table S1: Dietary References Intakes (DRI) used in Diet Quality Score (DQS) assessment; Table S2: Recommended Dietary Allowance (RDA) and Adequate Intakes (AI) used in Total Nutrient Intake (TNI) and Food Nutrient Index (FNI) assessment.
