# Supplementary material for: Does Self-Perceived Diet Quality Align with Nutrient Intake? A Cross-Sectional Study Using the Food Nutrient Index and Diet Quality Score
Source: Nutrients. 2023 Jun 12;15(12):2720. doi: 10.3390/nu15122720 (PMC10305402; doi:10.3390/nu15122720)
Supplement: Supplementary file 1 [file nutrients-15-02720-s001.zip › nutrients-2428849-supplementary.pdf]

Supplementary Table S1. Dietary References Intakes (DRI) used in Diet Quality Score (DQS) assessment.

Nutrient	Men				Women			
	**19–30 y**	**31–50 y**	**51–70 y**	**> 70 y**	**19–30 y**	**31–50 y**	**51–70 y**	**> 70 y**
Vitamin C	75 mg/d	75 mg/d	75 mg/d	75 mg/d	60 mg/d	60 mg/d	60 mg/d	60 mg/d
Vitamin E	12 mg/d	12 mg/d	12 mg/d	12 mg/d	12 mg/d	12 mg/d	12 mg/d	12 mg/d
Vitamin B1	1 mg/d	1 mg/d	1 mg/d	1 mg/d	0.9 mg/d	0.9 mg/d	0.9 mg/d	0.9 mg/d
Vitamin B2	1.1 mg/d	1.1 mg/d	1.1 mg/d	1.1 mg/d	0.9 mg/d	0.9 mg/d	0.9 mg/d	0.9 mg/d
Vitamin B3	12 mg/d	12 mg/d	12 mg/d	12 mg/d	11 mg/d	11 mg/d	11 mg/d	11 mg/d
Vitamin B6	1.1 mg/d	1.1 mg/d	1.4 mg/d	1.4 mg/d	1.1 mg/d	1.1 mg/d	1.3 mg/d	1.3 mg/d
Vitamin B12	2 mcg/d	2 mcg/d	2 mcg/d	2 mcg/d	2 mcg/d	2 mcg/d	2 mcg/d	2 mcg/d
Phosphorus	580 mg/d	580 mg/d	580 mg/d	580 mg/d	580 mg/d	580 mg/d	580 mg/d	580 mg/d
Magnesium	330 mg/d	350 mg/d	350 mg/d	350 mg/d	255 mg/d	265 mg/d	265 mg/d	265 mg/d
Selenium	45 mcg/d	45 mcg/d	45 mcg/d	45 mcg/d	45 mcg/d	45 mcg/d	45 mcg/d	45 mcg/d

DRI modified from [**Error! Reference source not found.**]. Recommended nutrient intakes for macronutrients as well as for vitamin A, iron and zinc were obtained from [**Error! Reference source not found.**]. The following recommended nutrient intakes were used for DQS scoring (based on [**Error! Reference source not found.**,**Error! Reference source not found.**]): Carbohydrate: 50–60% of total energy; fat: <30% of total energy; saturated fat: <10% of total energy; protein: 0.86 g/kg body weight; vitamin A: ≥625 mcg/d in men and ≥550 mcg/d in women; iron: ≥6 mg/d in men and ≥8.1 mg/d in women; zinc: ≥9.4 mg/d in men and ≥6.8 mg/d in women.

Supplementary Table S2. Recommended Dietary Allowance (RDA) and Adequate Intakes (AI) used in Total Nutrient Intake (TNI) and Food Nutrient Index (FNI) assessment.

Nutrient	Men			Women		
	**19–30 y**	**31–50 y**	**51–70 y**	**19–30 y**	**31–50 y**	**51–70 y**
Calcium	1000 mg/d	1000 mg/d	1000 mg/d	1000 mg/d	1000 mg/d	1200 mg/d
Magnesium	400 mg/d	420 mg/d	420 mg/d	310 mg/d	320 mg/d	320 mg/d
Potassium	3400 mg/d	3400 mg/d	3400 mg/d	2600 mg/d	2600 mg/d	2600 mg/d
Choline	425 mg/d	425 mg/d	425 mg/d	550 mg/d	550 mg/d	550 mg/d
Vitamin A	700 mcg RAE/d	700 mcg RAE/d	700 mcg RAE/d	900 mcg RAE/d	900 mcg RAE/d	900 mcg RAE/d
Vitamin C	75 mg/d	75 mg/d	75 mg/d	90 mg/d	90 mg/d	90 mg/d
Vitamin D	600 IU/d	600 IU/d	600 IU/d	600 IU/d	600 IU/d	600 IU/d
Vitamin E	15 mg/d	15 mg/d	15 mg/d	15 mg/d	15 mg/d	15 mg/d

RDA and AI found in [**Error! Reference source not found.**.]
